# The association of education with body mass index and waist circumference in the EPIC-PANACEA study

**DOI:** 10.1186/1471-2458-11-169

**Published:** 2011-03-17

**Authors:** Silke Hermann, Sabine Rohrmann, Jakob Linseisen, Anne M May, Anton Kunst, Herve Besson, Dora Romaguera, Noemie Travier, Maria-Jose Tormo, Esther Molina, Miren Dorronsoro, Aurelio Barricarte, Laudina Rodríguez, Francesca L Crowe, Kay-Tee Khaw, Nicholas J Wareham, Petra GA van Boeckel, H Bas Bueno-de-Mesquita, Kim Overvad, Marianne Uhre Jakobsen, Anne Tjønneland, Jytte Halkjær, Claudia Agnoli, Amalia Mattiello, Rosario Tumino, Giovanna Masala, Paolo Vineis, Androniki Naska, Philippos Orfanos, Antonia Trichopoulou, Rudolf Kaaks, Manuela M Bergmann, Annika Steffen, Bethany Van Guelpen, Ingegerd Johansson, Signe Borgquist, Jonas Manjer, Tonje Braaten, Guy Fagherazzi, Françoise Clavel-Chapelon, Traci Mouw, Teresa Norat, Elio Riboli, Sabina Rinaldi, Nadia Slimani, Petra HM Peeters

**Affiliations:** 1 Division of Cancer Epidemiology, German Cancer Research Centre, Heidelberg, Germany; 2 Institute of Epidemiology, Helmholtz Centre Munich, Neuherberg, Germany; 3 Julius Centre for Health Sciences and Primary Care, University Medical Centre Utrecht, Utrecht, The Netherlands; 4 National Institute for Public Health and the Environment (RIVM), Bilthoven, The Netherlands; 5 Academic Medical Centre (AMC), University of Amsterdam, Amsterdam, The Netherlands; 6 Medical Research Council, Epidemiology Unit, Institute of Metabolic Science, Cambridge, UK; 7 Department of Epidemiology & Public Health, Imperial College London, London, UK; 8 Unit of Nutrition, Environment and Cancer, Catalan Institute of Oncology, IDIBELL, Barcelona, Spain; 9 Epidemiology Service, Murcia Health Council, Murcia, Spain; 10 Preventive Medicine and Public Health Unit, Murcia Medical School, Murcia, Spain; 11 CIBER Epidemiología y Salud Pública (CIBERESP), Spain; 12 Andalusian School of Public Health, Granada, Spain; 13 Public Health Department of Gipuzkoa, San Sebastian, Spain; 14 Public Health Institute of Navarra, Pamplona, Spain; 15 Public Health and Participation Directorate, Health and Health Care Services Council, Asturias, Spain; 16 Cancer Epidemiology Unit, University of Oxford, Oxford, UK; 17 Department of Public Health and Primary Care, University of Cambridge, Cambridge, UK; 18 Department of Cardiology, Aalborg Hospital, Aarhus University Hospital, Aalborg, Denmark; 19 Department of Clinical Epidemiology, Aarhus University Hospital, Aalborg, Denmark; 20 Danish Cancer Society, Institute of Cancer Epidemiology, Copenhagen, Denmark; 21 Nutritional Epidemiology Unit, Fondazione IRCCS Istituto Nazionale dei Tumori, Milan, Italy; 22 Department of Clinical and Experimental Medicine - Federico II University, Naples, Italy; 23 Cancer Registry and Histopathology Unit, Department of Oncology, "Civile - M.P.Arezzo" Hospital, Ragusa, Italy; 24 Molecular and Nutritional Epidemiology Unit, Cancer Research and Prevention Institute (ISPO), Florence, Italy; 25 ISI Foundation, Torino, Italy; 26 Environmental Epidemiology, Imperial College London, London, UK; 27 Department of Hygiene and Epidemiology, University of Athens Medical School, Athens, Greece; 28 Hellenic Health Foundation, Greece; 29 German Institute of Human Nutrition Potsdam-Rehbrücke, Nuthetal, Germany; 30 Department of Medical Biosciences, Pathology, Umeå University, Umeå, Sweden; 31 Department of Odontology, Umeå University, Umeå, Sweden; 32 Department of Oncology, Lund University Hospital, Lund, Sweden; 33 Department of Surgery, Malmö University Hospital, Malmö, Sweden; 34 Institute of Community Medicine, University of Tromsø, Tromsø, Norway; 35 Inserm ERI20 and Paris South University, Institut Gustave-Roussy, Villejuif, France; 36 International Agency for Research on Cancer, Lyon, France; 37 Insitute of Social and Preventive Medicine, University of Zurich, Zurich, Switzerland

**Keywords:** socioeconomic status, education, BMI, waist circumference, cohort study, EPIC

## Abstract

**Background:**

To examine the association of education with body mass index (BMI) and waist circumference (WC) in the European Prospective Investigation into Cancer and Nutrition (EPIC).

**Method:**

This study included 141,230 male and 336,637 female EPIC-participants, who were recruited between 1992 and 2000. Education, which was assessed by questionnaire, was classified into four categories; BMI and WC, measured by trained personnel in most participating centers, were modeled as continuous dependent variables. Associations were estimated using multilevel mixed effects linear regression models.

**Results:**

Compared with the lowest education level, BMI and WC were significantly lower for all three higher education categories, which was consistent for all countries. Women with university degree had a 2.1 kg/m^2 ^lower BMI compared with women with lowest education level. For men, a statistically significant, but less pronounced difference was observed (1.3 kg/m^2^). The association between WC and education level was also of greater magnitude for women: compared with the lowest education level, average WC of women was lower by 5.2 cm for women in the highest category. For men the difference was 2.9 cm.

**Conclusion:**

In this European cohort, there is an inverse association between higher BMI as well as higher WC and lower education level. Public Health Programs that aim to reduce overweight and obesity should primarily focus on the lower educated population.

## Background

Overweight and obesity are growing problems worldwide with a prevalence of overweight and obesity of 60% for European women and 70% for men in the age group of 45-59 years [1]. Being overweight or obese increases the risk of some types of cancer, cardiovascular disease, hypertension, diabetes mellitus type 2, gallstones, osteoarthritis, or sleep apnea [2]. In most Western countries, there is a clear association between socioeconomic status (SES) and the risk of becoming overweight or obese as pointed out by McLaren [3]. Data from NHANES 1999/2000 survey have shown a higher prevalence of obesity in low educated men and women compared with high educated subjects, although the difference between these groups decreased between the survey in the early 1970s and the 1999/2000 survey [4]. In the WHO MONICA project, years of schooling and BMI were also significantly inversely associated [5]. In contrast to the US results, MONICA results indicate an increase in the gap between obesity in less and better educated subjects in most of the participating centers. It is interesting to note that in both surveys a trend towards a higher education in the survey populations has been observed.

Although body mass index (BMI) is the most commonly used anthropometric measure of obesity, other measures such as waist circumference (WC) are increasingly being used. WC is of special interest since previous evaluations of the European Prospective Investigation into Cancer and Nutrition (EPIC) have shown that WC was stronger related to overall mortality than BMI [6]. EPIC-PANACEA (Physical Activity, Nutrition, Alcohol, Cessation of smoking, Eating out of home And obesity) offers the opportunity to evaluate the association between highest educational level attained and measurements of BMI and WC in a large European population.

## Methods

### Population and study design

EPIC is an ongoing multi-centre prospective cohort study consisting of 23 centres in 10 countries (Denmark, France, Germany, Greece, Italy, the Netherlands, Norway, Spain, Sweden and the United Kingdom). From 1992 to 2000, more than 500,000 individuals (in majority 35 to 70 years of age) were recruited from the population living in a defined geographical region. Recruitment procedures have been described in detail by Riboli et al. [7]. The cohort of France is based on female members of a health insurance plan for school employees; parts of the Italian and Spanish cohorts included members of local blood donors associations; the cohorts from Utrecht (The Netherlands) and Florence (Italy) recruited participants of breast cancer screening programs; and the Oxford cohort consisted of vegetarians, vegans and other health-conscious individuals. In France, Norway, Utrecht (The Netherlands) and Naples (Italy) only women were recruited [7]. Baseline information on education, occupation, medical history, tobacco smoking, physical activity and reproductive history were assessed using questionnaires and/or interviews. Usual diet was measured by country-specific assessment instruments. Seven countries adopted an extensive self-administered dietary questionnaire. In Greece, Spain and Ragusa a dietary questionnaire was administered by direct interview. A food frequency questionnaire and a seven-day record were adopted in the UK. In Malmö, Sweden, a quantitative questionnaire combined with a 7-day menu book and an interview was used [7]. Approval for this study was obtained from the ethical review boards of all participating institutions.

Of the total cohort of 519,931 apparently healthy subjects, we excluded subjects with missing information on dietary and non-dietary variables (n = 6,675), BMI (n = 4,011), or education (n = 20,170), subjects with an extreme ratio of energy intake to energy expenditure (n = 10,209), pregnant women (n = 623), and subjects with implausible anthropometric measurements (n = 376). The analytical cohort consisted of 141,230 men and 336,637 women.

### Anthropometric measurements

In most EPIC centres height and weight were measured at recruitment following a standardized procedure and is described in detail elsewhere [8]. In France, Oxford and Norway, self-reported data were obtained from all individuals. For part of the Oxford (UK) cohort, for which measured data were not available, linear regression models were used to predict sex- and age-specific values from subjects with both measured and self-reported body measures [9,10]. In each centre, WC was measured either at the narrowest torso circumference or midway between the lower ribs and the iliac crest. To reduce heterogeneity due to protocol differences in clothing worn during measurement, correction factors of - 1.5 kg for weight and - 2.0 cm for WC were adopted for subjects who were normally dressed and without shoes, while an adjustment for weight of - 1.0 kg was applied for subjects in light clothing [8]. While BMI information (measured or self-reported) was available for all subjects, WC measurements were only available for 73% of the subjects as waist circumference has not been measured in Norway, Umea (Sweden), and in the majority of the French cohort.

BMI was calculated as weight (kg) divided by height (m) squared. We used the following BMI categories: < 18.5 kg/m^2^, underweight; BMI ≥ 18.5 to < 25 kg/m^2^, normal weight; BMI ≥ 25 to < 30 kg/m^2^, overweight; BMI ≥ 30 kg/m^2^, obese.

### Highest Level of Education

Educational level, based on highest school level reached (university, secondary, technical or professional, primary, or none), was used as a proxy for SES. This variable was categorized into: (1) primary school or less; (2) vocational secondary education; (3) other secondary education; and (4) university degree.

### Covariates

Recruitment age, smoking, physical activity, alcohol consumption, total energy intake and marital status were taken into account as co-variables. Smoking status was categorized as current, former, never and missing. To adjust for the level of physical activity, a five-level validated variable (inactive, moderately inactive, moderately active, active, and missing) was created [11]. Information on alcohol consumption reflected the amount of alcohol consumed daily during the 12 months prior to recruitment. This information was summarized in a six-level variable for women (non consumers, 1-6, 7-18, 19-30, 31-60, > 60 g/day) and a seven-level variable for men (non consumers, 1-6, 7-18, 19-30, 31-60 g/day, 61-96, > 96 g/day). Total energy intake was computed from the dietary assessment instruments. Marital status was categorised as single/separated/widowed, living together/married and missing.

### Statistical methods

The associations between BMI, WC and education were examined for the total EPIC cohort and by country. All analyses were carried out by sex. The association between education and BMI or WC across all countries was estimated using multilevel mixed linear models with random intercepts and coefficients both at the centre and country level. The analysis by countries was done depending on the number of study centres per country. For countries with only one centre (i.e., the Netherlands [men], France, Norway, and Greece), adjusted linear models were run. For countries with more than one study centre (i.e., Italy, Spain, the Netherlands [women], Sweden, Denmark, Germany, and United Kingdom,), adjusted mixed linear models with random intercept at centre level were used to assess the association between highest education level and BMI/WC.

In all models, BMI and WC were modelled as continuous variables. Education level was the independent variable and modelled using a categorical variable. Age at recruitment and total energy intake were entered in the models as continuous variables while physical activity, smoking, and alcohol consumption were entered in the models as categorical variables. Further adjusting for marital status did not change our results and was not included in the final models. Secondary analyses were performed by age group (age at recruitment </≥ 60 years), smoking status, categories of alcohol consumption (0-<6/≥6 g/day), as well as by BMI (</≥25 kg/m2) and WC (</≥88 cm in women; </≥102 cm in men [12]). All statistical analyses were performed with SAS software version 9.1 (SAS Institute, Cary, NC, USA).

## Results

The distribution of educational levels varies widely in the EPIC cohort (Table 1). The percentage of men having only completed primary school ranged from 10.9% (Dutch cohorts) to 38.7% (Spanish centers); in women, the country with the lowest percentage of subjects that have only completed primary school was in the French cohort, which consists of female school employees (11.1%) and highest in the Spanish cohorts (41.8%). In the Italian cohorts, 14.4% of men had a university degree compared to 42.5% in the two German cohorts; in women, the lowest percentage of women with university degree was observed in the Spanish cohorts (10.0%) and the highest in the British cohorts (39.5%). Besides the Greek and the Spanish cohorts, only few study participants fell into the category with no formal educational degree. Therefore, we had combined the categories "no degree" and "primary school completed" into "primary school or less".

**Table 1:** Distribution of EPIC participants by sex, country, and highest level of education attained

		Men					Women				
		**Educational Level**					**Educational Level**				
		**1**	**2**	**3**	**4**	**Total**	**1**	**2**	**3**	**4**	**Total**

France	n	--	--	--	--	--	7944	--	35437	25699	69080
	%	--	--	--	--	--	11.5	--	51.3	37.2	
Italy	n	2426	2130	7626	2041	14223	9270	3461	14317	4172	31220
	%	17.1	15.0	53.6	14.4		29.7	11.1	45.9	13.4	
Spain	n	9308	1952	1206	2232	14698	18651	1375	1390	2385	23801
	%	63.3	13.3	8.2	15.2		78.4	5.8	5.8	10.0	
United Kingdom	n	3214	6514	2514	7827	20069	5457	14471	7152	17699	44779
	%	16	32.5	12.5	39.0		12.2	32.3	16.0	39.5	
The Netherlands	n	1093	4136	2079	2681	9989	5213	9425	8797	5253	28688
	%	10.9	41.4	20.8	26.8		18.2	32.9	30.7	18.3	
Greece	n	5393	1962	1581	1718	10654	9557	1007	2891	1816	15271
	%	50.6	18.4	14.8	16.1		62.6	6.6	18.9	11.9	
Germany	n	5512	6137	1156	9446	22251	7017	12260	2316	7833	29426
	%	24.8	27.6	5.20	42.5		23.9	41.7	7.9	26.6	
Sweden	n	8460	4930	4790	4649	22829	10064	7715	4685	6982	29446
	%	37.1	21.6	20.98	20.4		34.2	26.2	15.9	23.7	
Denmark	n	9193	7769	2054	7501	26517	9128	13568	3455	2996	29147
	%	34.7	29.3	7.8	28.3		31.3	46.6	11.9	10.3	
Norway	n	--	--	--	--	--	8206	12800	10306	4467	35779
	%	--					22.9	35.8	28.8	12.5	

Total	n	44599	35530	23006	38095	141230	90507	76082	90746	79302	336637

1 = no formal degree or primary school completed ("primary school or less"); 2 = vocational secondary training; 3 = other secondary education; 4 = university

Baseline characteristics of the study participants are shown in Table 2. Subjects with a low educational level were oldest at time of recruitment, had the highest prevalence of overweight and obesity of all education categories, and reported the lowest level of physical activity. Men and women with a university degree were less often current smokers than participants who were less educated. Women with the lowest education also had the lowest alcohol consumption.

**Table 2:** Baseline Characteristics of EPIC participants by sex and highest level of education; 1992-2000

	Men				Women			
	Primary school or less	Vocational secondary education	Other secondary education	University	Primary school or less	Vocational secondary education	Other secondary education	University
n (%)	44599 (31.6)	35530 (25.2)	23006 (16.3)	38095 (27.0)	90507 (26.9)	76082 (22.6)	90746 (27.0)	79302 (23.6)

	*Median (interquartile range)*							
Age at recruitment (years)	56.7 (50.6-61.8)	52.1 (45.5-58.5)	48.7 (40.4-56.0)	51.4 (43.5-57.7)	54.7 (48.7-60.9)	51.1 (44.4-56.7)	50.2 (44.5-56.1)	48.3 (42.9-54.3)
Total energy intake (kcal/day)	2381 (1954-2877)	2341 (1938-2806)	2439 (2008-2931)	2304 (1927-2730)	1823 (1498-2209)	1807 (1508-2157)	1958 (1620-2355)	1935 (1608-2311)
Alcohol consumption at baseline (g/d)	12.6 (3.0-32.6)	12.8 (4.2-29.4)	12.1 (3.7-28.1)	15.0 (6.1-30.9)	1.5 (0.0-7.3)	3.8 (1.0-10.6)	4.0 (0.7-11.9)	6.2 (1.6-13.8)
BMI (kg/m^2^)	27.2 (24.9-29.7)	26.1 (24.1-28.4)	25.7 (23.6-28.0)	25.4 (23.4-27.6)	26.3 (23.6-29.8)	24.3 (22.1-27.2)	23.3 (21.3-25.8)	22.7 (20.9-25.1)
WC (cm)	97.0 (91.0-104.0)	94.0 (87.5-100.0)	92.3 (86.3-99.0)	92.0 (86.0-98.0)	85.0 (77.0-93.0)	77.5 (71.2-85.3)	77.0 (71.0-84.0)	74.0 (69.0-80.8)
	*Percent*							
Prevalence of overweight (%)^a^	51.8	50.0	46.5	45.1	38.2	31.2	24.4	20.1
Prevalence of obesity (%)^a^	22.5	14.2	12.1	9.9	23.9	11.7	7.4	5.4
Smoking status								
Never	27.4	30.2	34.8	40.8	60.9	46.5	56.4	56.3
Former	37.0	37.9	34.6	35.7	16.3	26.8	22.5	26.2
Smoker	34.6	31.1	29.5	22.6	21.3	25.4	18.2	15.2
Missing	1.0	0.8	1.1	0.9	1.5	1.2	3.0	2.4
Physical activity								
Inactive	20.5	14.8	14.9	16.3	33.6	13.9	18.0	14.1
Moderately inactive	23.7	25.6	28.7	35.1	27.8	27.9	32.3	34.2
Moderately active	22.4	22.0	19.4	23.0	14.4	18.5	22.3	27.0
Active	24.8	26.8	19.7	18.4	10.7	17.9	11.3	14.5
Missing	8.6	10.8	17.2	7.3	13.6	21.8	18.2	10.2
Marital status								
Single/divorced/separated/widowed	9.4	13.2	15.9	15.8	12.8	15.8	17.0	24.9
Married/living together	48.3	58.4	65.8	58.3	54.5	61.5	74.8	66.5
Missing	42.3	28.4	18.3	25.9	32.7	22.7	8.2	8.6

^a ^overweight defined as BMI ≥ 25 and < 30, obesity defined as BMI ≥ 30

^b ^does not add up to 100% due to missing information

Compared to women with lowest education, women with a university degree had a 2.12 kg/m^2 ^lower BMI (Table 3). For men, result was similar although less pronounced (1.28 kg/m^2^). Crude results were similar compared with fully adjusted models. The difference between lowest and highest education group was larger in younger than in older women. The difference in BMI was also stronger in younger men, but less pronounced than in women. In women, the difference between highest and lowest educational group was stronger in never than in current smokers, but the confidence intervals were wide and overlapping. We observed strongly attenuated associations of education with BMI in non-obese subjects. In women, but not in men, the difference between highest and lowest education status was still statistically significant in non-obese subjects, but the difference was merely 0.5 BMI units.

**Table 3:** Association^a,b ^between level of education and BMI (kg/m^2^) in EPIC by sex and subgroups; EPIC participants interviewed between 1992 and 2000

	Primary school or less	Vocational secondary training		Other secondary education		University	
		Estimate	95% CI	Estimate	95% Ci	Estimate	95% CI
**BMI (kg/m^2^)**							
**Women**							
Overall crude	ref.	-1.16	-2.46 to 0.14	-1.58	-2.69 to -0.47	-2.25	-3.39 to -1.10
Overall adjusted^a^	ref.	-0.98	-1.11 to -0.85	-1.44	-1.69 to -1.20	-2.12	-2.49 to -1.76
Age > = 60	ref.	-0.84	-0.98 to -0.70	-1.25	-1.47 to -1.03	-1.56	-1.88 to -1.24
Age < 60	ref.	-1.30	-1.56 to -1.04	-1.46	-1.72 to -1.20	-2.13	-2.48 to -1.78
Never smoker	ref.	-1.19	-3.45 to 1.08	-1.68	-3.70 to 0.35	-2.37	-4.42 to -0.32
Former smoker	ref.	-1.03	-2.16 to 0.10	-1.51	-2.56 to -0.45	-2.04	-3.08 to -0.99
Current smoker	ref.	-0.90	-1.52 to -0.29	-1.05	-1.65 to -0.45	-1.59	-2.23 to -0.95
Alcohol intake 0- < 6 g/day	ref.	-1.10	-2.40 to 0.20	-1.54	-2.67 to -0.41	-2.23	-3.38 to -1.08
Alcohol intake ≥ 6 g/day	ref.	-1.05	-2.14 to 0.04	-1.42	-2.35 to -0.50	-1.97	-2.91 to -1.03
BMI < 25 kg/m^2^	ref.	-0.15	-0.65 to 0.35	-0.27	-0.72 to 0.19	-0.47	-0.92 to -0.02
BMI ≥ 25 kg/m^2^	ref.	-0.57	-1.16 to 0.01	-0.76	-1.25 to -0.27	-1.08	-1.64 to -0.53
							
**Men**							
Overall crude	ref.	-0.56	-1.68 to 0.56	-0.81	-1.91 to 0.29	-1.28	-2.45 to -0.10
Overall adj.	ref.	-0.52	-0.61 to -0.44	-0.84	-1.00 to -0.69	-1.28	-1.50 to -1.07
Age > = 60	ref.	-0.61	-0.79 to -0.44	-0.70	-0.90 to -0.49	-0.97	-1.16 to -0.77
Age < 60	ref.	-0.55	-0.68 to -0.43	-0.84	-1.01 to -0.67	-1.36	-1.55 to -1.18
Never smoker	ref.	-0.66	-1.27 to -0.06	-0.95	-1.59 to -0.31	-1.52	-2.23 to -0.82
Former smoker	ref.	-0.63	-0.99 to -0.27	-0.83	-1.24 to -0.42	-1.28	-1.80 to -0.75
Current smoker	ref.	-0.50	-1.67 to 0.67	-0.85	-2.03 to 0.33	-1.14	-2.34 to 0.05
Alcohol intake 0- < 6 g/day	ref.	-0.67	-1.83 to 0.49	-0.89	-2.06 to 0.28	-1.42	-2.68 to -0.15
Alcohol intake ≥ 6 g/day	ref.	-0.56	-1.16 to 0.04	-0.87	-1.46 to -0.28	-1.33	-2.02 to -0.64
BMI < 25 kg/m^2^	ref.	-0.01	-0.16 to 0.15	-0.02	-0.20 to 0.15	-0.04	-0.24 to 0.15
BMI ≥ 25 kg/m^2^	ref.	-0.52	-0.79 to -0.24	-0.62	-0.88 to -0.37	-0.95	-1.25 to -0.66

^a^adjusted for recruitment age, smoking, physical activity, alcohol consumption, total energy intake (when applicable)

^b^the association between education and BMI or WC across all countries was estimated using multilevel mixed linear models with random intercepts and coefficients both at the centre and country level.

**Figure 1:**
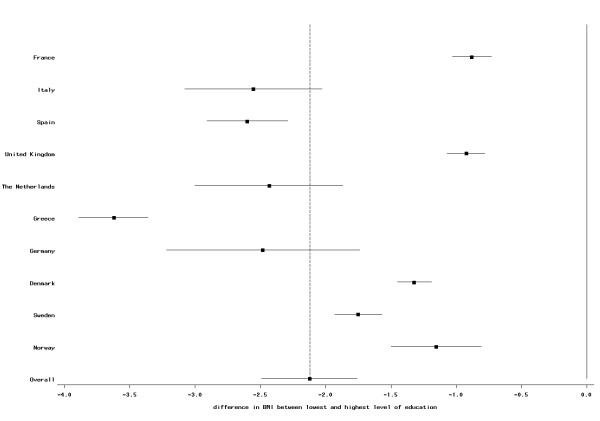
**Difference (mean and 95% CI) in BMI (in kg/m^2^) between highest and lowest educational level in women; EPIC participants interviewed between 1992 and 2000**. The dotted vertical line indicates the overall mean difference between highest and lowest educational level.

**Figure 2:**
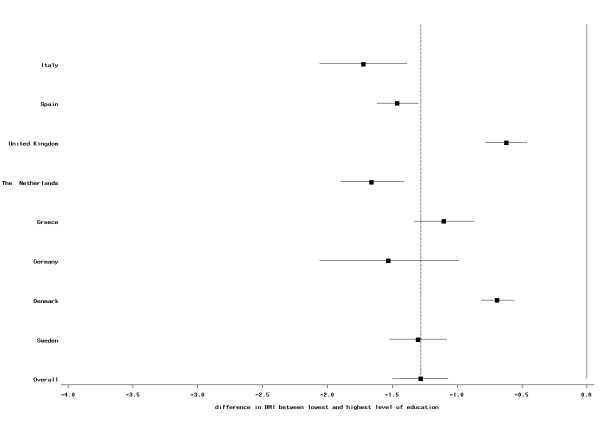
**Difference (mean and 95% CI) in BMI (in kg/m^2^) between highest and lowest educational level in men; EPIC participants interviewed between 1992 and 2000**. The dotted vertical line indicates the overall mean difference between highest and lowest educational level.

The direction of the overall association between BMI and education was consistent in all countries, although the strength of the association differed between countries. In women the association was weakest in the French cohort and strongest in the Greek cohort (Figure 1). In men, the weakest association was observed in the British centers, while the association was most pronounced in the Italian centers (Figure 2). For all countries, but men of the Greek and Danish cohorts there was a clear trend between level of education and BMI; however, in all countries, BMI was significantly lower for all three higher education categories compared with the lowest education level (data not shown).

The association between WC and education level was stronger for women than for men: compared with the lowest education level, the average waist circumference was statistically significantly lower by 5.20 cm for female participants in the highest category (Table 4). For men the respective difference was 2.94 cm. Crude associations were similar to the fully adjusted models. Age stratification revealed a stronger difference in WC with education in elderly men compared to younger men. However, for women the difference was larger in the younger than in the older age group. As seen for BMI, the difference between highest and lowest educational group was stronger in never than in current smokers, but again with wide and overlapping confidence intervals. The observed differences were similar between non- or occasional consumers of alcoholic beverages and regular consumers (≥6 g ethanol/day). Even among women with a waist circumference < 88 cm, the difference between highest and lowest educated women was statistically significant, but not among men with normal waist (< 102 cm). When adding BMI to the statistical model, all associations for WC were attenuated and lost statistical significance (data not shown).

**Table 4:** Association^a,b ^between level of education and waist circumference (cm) in EPIC by sex and *subgroups*; EPIC participants interviewed between 1992 and 2000

	Primary school or less	Vocational secondary education		Other secondary education		University	
		Estimate	95% CI	Estimate	95% Ci	Estimate	95% CI
**Waist (cm)**							
**Women**							
Overall crude	ref.	-3.23	-5.72 to -0.74	-3.98	-6.10 to -1.87	-5.43	-7.76 to -3.10
Overall adj.	ref.	-2.62	-2.94 to -2.30	-3.71	-4.32 to -3.10	-5.20	-6.10 to -4.30
Age > = 60	ref.	-2.06	-2.54 to -1.58	-3.02	-3.49 to -2.56	-3.83	-4.74 to -2.91
Age < 60	ref.	-3.39	-3.99 to -2.80	-4.09	-4.57 to -3.62	-5.47	-6.19 to -4.76
Never smoker	ref.	-3.66	-5.84 to -1.48	-4.44	-6.38 to -2.50	-5.85	-7.98 to -3.72
Former smoker	ref.	-2.94	-4.96 to -0.91	-3.73	-5.77 to -1.70	-5.06	-7.05 to -3.07
Current smoker	ref.	-2.69	-3.85 to -1.54	-2.88	-4.04 to -1.72	-4.11	-5.29 to -2.92
Alcohol intake 0- < 6 g/day	ref.	-3.21	-5.59 to -0.83	-3.95	-6.02 to -1.89	-5.41	-7.73 to -3.09
Alcohol intake ≥ 6 g/day	ref.	-3.37	-5.07 to -1.68	-4.02	-5.68 to -2.37	-5.19	-6.84 to -3.54
waist circumf. < 88 cm	ref.	-1.28	-2.57 to 0.01	-1.65	-2.91 to -0.40	-2.25	-3.51 to -0.98
waist circumf. ≥ 88 cm	ref.	-0.85	-1.47 to -0.23	-1.31	-1.70 to -0.91	-1.63	-2.32 to -0.94
							
**Men**							
Overall crude	ref.	-1.49	-3.28 to 0.30	-1.75	-3.58 to 0.07	-2.84	-4.90 to -0.78
Overall adj.	ref.	-1.25	-1.50 to -1.01	-1.97	-2.41 to -1.54	-2.94	-3.55 to -2.33
Age > = 60	ref.	-1.44	-1.95 to -0.93	-1.53	-2.07 to -.99	-2.15	-2.70 to -1.61
Age < 60	ref.	-1.39	-1.71 to -1.08	-1.96	-2.39 to -1.53	-1.96	-3.63 to -2.67
Never smoker	ref.	-1.93	-3.64 to -0.22	-2.29	-4.03 to -0.55	-3.70	-5.60 to -1.80
Former smoker	ref.	-1.51	-3.17 to 0.14	-1.92	-3.71 to -0.14	-3.06	-4.99 to -1.12
Current smoker	ref.	-1.33	-4.66 to 2.01	-2.08	-5.48 to 1.32	-2.51	-5.88 to 0.86
Alcohol intake 0- < 6 g/day	ref.	-1.67	-4.95 to 1.61	-2.05	-5.50 to 1.40	-3.26	-6.78 to 0.26
Alcohol intake ≥ 6 g/day	ref.	-1.53	-3.26 to 0.21	-2.15	-3.88 to -0.42	-3.14	-5.07 to -1.22
Waist circumf. < 102 cm	ref.	-0.40	-1.61 to 0.82	-0.76	-2.10 to 0.57	-1.29	-2.67 to 0.09
Waist circumf. ≥ 102 cm	ref.	-0.60	-0.98 to -0.22	-0.54	-0.99 to -0.10	-1.03	-1.40 to -0.66

^a^adjusted for recruitment age, smoking, physical activity, alcohol consumption, total energy intake (when applicable)

^b^the association between education and BMI or WC across all countries was estimated using multilevel mixed linear models with random intercepts and coefficients both at the centre and country level

**Figure 3:**
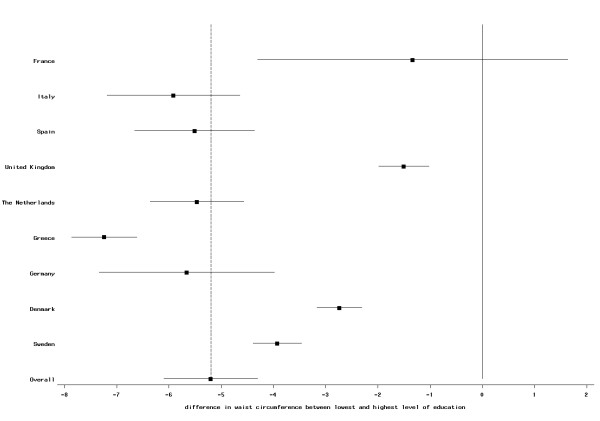
**Difference (mean and 95% CI) in waist circumference (in cm) between highest and lowest educational level in women; EPIC participants interviewed between 1992 and 2000**. The dotted vertical line indicates the overall mean difference between highest and lowest educational level.

**Figure 4:**
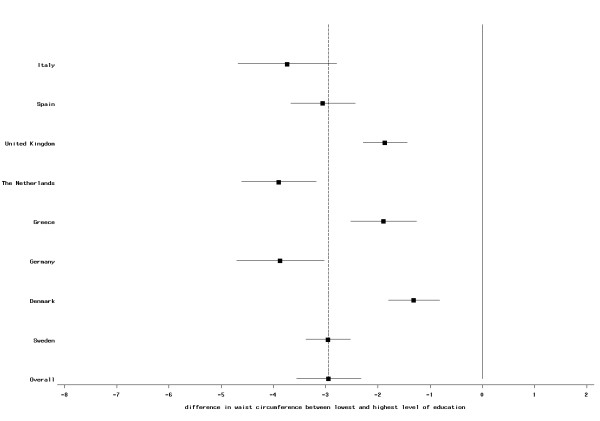
**Difference (mean and 95% CI) in waist circumference (in cm) between highest and lowest educational level in men; EPIC participants interviewed between 1992 and 2000**. The dotted vertical line indicates the overall mean difference between highest and lowest educational level.

These associations were observed in most countries, but the magnitude of the effect differed between countries. In females, the association was weakest in the British centers and strongest in women of the Greek cohort; no statistically significant difference was observed in French women (Figure 3). In almost all centers besides France, women with secondary school or technical/professional school also had significant lower waist circumference compared to women with low education. For men, the relation was smallest in the Danish cohorts and strongest in the Dutch centers (Figure 4). Men of the Greek and the Swedish cohorts had a non-significant difference in waist circumference in participants with secondary school and technical/professional school; for all other centers, the difference was statistically significant (data not shown).

## Discussion

WC is a measure of central adiposity, while BMI is generally considered as an indicator of overall obesity. In this European cohort, we observed that higher educated participants had lower BMI and as well as smaller WC. However, when adjusting WC for BMI, the association of education with WC was strongly attenuated, indicating that BMI is a good indicator of the association between education and obesity.

This inverse association between BMI and educational level is in line with results in other studies [2,3,13-15], some also showing a stronger association for women than for men [3,5,16,17]. However, the reason for this difference is still mostly unclear. Differences between SES categories in physical activity and energy intake might explain part of the association between SES and BMI [18], but this is not observed in our and other studies [19]. Furthermore, it could not be shown that SES status affects either total energy intake or macronutrients composition of the diet [20]. Similarly, in EPIC total energy intake did not differ strongly between the education categories (see Table 2). Another explanation is that underreporting might be more common in less educated subjects. Individuals with a higher BMI as well as those who want to reduce weight tend to underreport dietary intake to a greater degree than individuals with lower BMI [21-23]. This behaviour seems to be more common among women than among men in EPIC [24]. Since 74% of the subjects in the lowest education category are either overweight or obese, the impact of dietary underreporting may be more meaningful among less educated people. The observed inverse SES gradients in BMI and WC are, thus, likely underestimated. Furthermore, it can be speculated that foods with a high energy density and an unhealthy image are underreported. Energy expenditure is a further important factor that influences BMI. Subjects in the lowest education level stated to be inactive most frequently (22.4% of men and 38.9% of females). It has also been shown that individuals who overestimated energy expenditure on the physical activity records had a significantly higher BMI and percentage of body fat compared with those that accurately estimated their energy expenditure [25,26].

Overall, we observed a difference in BMI of 2.12 kg/m^2 ^in women and of 1.28 kg/m^2 ^in men when comparing highest with lowest educational level. Although Molarius et al. [5,27] did not estimate an overall difference in the MONICA surveys, our results are comparable with the MONICA results in range. It is interesting to note that the association between education and BMI was smallest in women from the Scandinavian centers as well as the UK cohort and the French. However, for France this could be explained by the relative homogenous SES level at study intake, because only teachers and other school employees were recruited. So, although at younger age the educational level might have differed, later on inequalities in SES disappeared. The association was strongest in Greece, but associations in the Spanish and Italian cohorts were more comparable to associations in centers from Middle Europe. Recently, Roskam et al. [27] showed that educational inequalities in overweight and obesity were largest in Mediterranean women, whereas they were largest in French, German, Belgian, and Czech women in the MONICA surveys [5]. For men, the inequalities are in general smaller and no clear geographical pattern emerge for Southern, Central, and Northern Europe [5,27]. In our analysis, it has to be taken into account, that although most cohorts were recruited from the general population, the cohorts are in the majority not representative of a country. Furthermore, as some cohorts have been recruited from specific subgroups of the population such as blood donors comparisons between the cohorts should be interpreted with caution.

Our study includes a large sample size and participants from ten European countries. However, for some centers, i.e., France, Oxford, and Norway, only self-reported information was available. Assuming an underreporting of weight and WC in these centers that is stronger in less than better educated individuals, this would cause a weaker association between BMI and WC and SES compared with other centers. This is what we indeed observed (Figures 1, 2, 3 and 4), although we still observed statistically significant relations between BMI and education in these centers. Differences in measurement of waist circumference between centers might also partly explain differences in the association between waist circumference and education between the centers. The EPIC participants were recruited over a time period of eight years (from 1992 to 2000). Changes in the prevalence of obesity and changes in the structure of the educational system (i.e., a trend towards a higher education in the general population) might lead to a small cohort effect, such that the association between SES and BMI could be different between subjects that have been recruited at the beginning of this period and subjects that have been recruited towards the end. Our data was too limited to study this.

Education was used in our analysis as an indicator of SES. Low educational levels may influence obesity-related behaviour such as diet and physical activity, which may be caused by lack of knowledge [28]. Compared to occupation and income, education is stable throughout life and reflects childhood conditions. However, stability can be a limitation because it does not take social advancements and status later in life into account [29]. In addition, SES of the spouse may be important, too. Neglecting this may result in an error that is probably more severe in older women, who adapted the SES of their partners after marriage. This may also explain the stronger effect seen in younger subjects (< 60 years of age). However, adjusting for marital status did not change our study results. Further variables to better capture a subject's SES such as household income have not consistently been assessed in all EPIC centers. The fact that the educational systems are diverse in the various European countries may lead to further misclassification. However, the lowest (primary school or less) as well as the highest educational level (university degree) should be rather comparable for all countries. Also, efforts have been made to correct for misclassification by comparing highest school level with years of schooling.

## Conclusion

In all European EPIC cohorts, there was an inverse association seen between BMI as well as WC and education level. Our results confirm previous literature on SES and BMI; as well add new information for the association between WC and level of education.

Public Health Programs that aim to reduce overweight and obesity should primarily focus on the lower educated population, such that these programs are better targeted to the addressed population group.

## Competing interests

The authors declare that they have no competing interests.

## Authors' contributions

PHMP: principal investigator of the EPIC-PANACEA project and guarantor of the article; ER: overall coordinator of the EPIC study, which was conceptualized, designed, and implemented in collaboration with the main investigators in the collaborating countries as follows: Denmark (KO and A Tj), France (FC-C), Germany (RK), Greece (ATr), Italy (RT, and PV), Netherlands (HBB-d-M and PHMP), Spain (LR, M-JT, MD, and AB), Sweden (JM), and United Kingdom (NW and K-TK) (these authors contributed to the study design, subject recruitment, and data collection and acquisition and are responsible for the ongoing follow-up and management of the EPIC cohort); SH, SRo and JL: conceived the current study; SH and SRo: responsible for the design of the study, analyses of data, interpretation of results; SH drafting of the manuscript, taking into account the comments and suggestions of the coauthors; contributors from the collaborating centers (AMM, AK, HB, DR, NT, EM, LR, FLC, PGAvB, MUJ, JH, CA, AM, GM, PO, AN, MMB, AS, BvG, IJ, SB, TB, GF, TM, TN, SRi and NS): provided the original data, information on the respective populations, and advice on study design, analysis, and interpretation of the results; and all coauthors: had the opportunity to comment on the analysis and interpretation of the findings and approved the final version of the manuscript.

## Pre-publication history

The pre-publication history for this paper can be accessed here:

http://www.biomedcentral.com/1471-2458/11/169/prepub
